# Biochemical Analyses of Sorghum Varieties Reveal Differential Responses to Drought

**DOI:** 10.1371/journal.pone.0154423

**Published:** 2016-05-06

**Authors:** Chukwuma C. Ogbaga, Piotr Stepien, Beth C. Dyson, Nicholas J. W. Rattray, David I. Ellis, Royston Goodacre, Giles N. Johnson

**Affiliations:** 1 The University of Manchester, Faculty of Life Sciences, Oxford Road, Manchester, United Kingdom; 2 Wroclaw University of Environmental and Life Sciences, Department of Plant Nutrition, ul. Grunwaldzka, Wroclaw, Poland; 3 Manchester Institute of Biotechnology, School of Chemistry, University of Manchester, United Kingdom; Institute of Genetics and Developmental Biology, Chinese Academy of Sciences, CHINA

## Abstract

We have examined the biochemical responses of two sorghum cultivars of differing drought tolerance, Samsorg 17 (more drought tolerant) and Samsorg 40 (less drought tolerant), to sustained drought. Plants were exposed to different degrees of drought and then maintained at that level for five days. Responses were examined in terms of metabolic changes and the expression of drought induced proteins—Heat Shock Proteins (HSPs) and dehydrins (DHNs). Generalised phenotypic changes were studied using Fourier transform infrared (FT-IR) Spectroscopy and non-targeted Gas Chromatography Mass Spectrometry (GC-MS) was employed to detect changes in metabolites, while changes in protein expression were examined using Western blot analysis. Different response profiles of metabolites, HSPs and DHNs were observed in the two cultivars. Metabolic changes involved variation in amino acids, polysaccharides and their derivatives. A total of 188 compounds, with 142 known metabolites and 46 unknown small molecules, were detected in the two sorghum varieties. Under water deficit conditions, Samsorg 17 accumulated sugars and sugar alcohols, while in Samsorg 40 amino acids increased in concentration. This study suggest that the two Sorghum varieties adopt distinct approaches in response to drought, with Samsorg 17 being better able to maintain leaf function under severe drought conditions.

## Introduction

Drought is one of the major limitations on food production especially in developing countries in Africa and Asia [1]. There is a growing need to produce crops that can survive extreme environmental conditions such as drought in order to maximise crop production. Detrimental effects of water deficiency can be seen in the roots, shoots and leaves of green plants at morphological, physiological and biochemical levels. In this paper, the emphasis will be on the plant’s biochemical responses to drought, in terms of its metabolism and quantification of selected proteins.

Exposure to drought triggers a variety of biochemical responses in plants and these responses include the accumulation of a variety of different compatible solutes and up-regulation of specific drought-induced proteins e.g. heat shock proteins (HSPs) and dehydrins (DHNs). Compatible solutes are osmolytes that protect plants from osmotic stress whilst maintaining cytosolic osmotic balance during extreme conditions in plants [2]. Drought has been reported to induce the accumulation of compatible solutes in various herbaceous plants [3–6]. Compatible solute accumulation has been described as either being a sign of stress or induced as a protective mechanism, which can minimise the effects of stress [2, 7–9]. On the one hand, their accumulation, enhanced by the up-regulation of abscisic acid (ABA), is seen as a plant’s signalling mechanism that can potentially mitigate the production of reactive oxygen species (ROS) [8], whilst on the other, their accumulation is thought to contribute to the maintenance of the cytosolic energy balance of a cell [9]. Examples of compatible solutes that have been identified as drought sensitive include nitrogen-containing compounds–e.g. glycine betaine, proline–and sugars–e.g. trehalose, glucose and sucrose [2, 10, 11]. These osmolytes are metabolically expensive to produce and, in the case of amino acids, represent an important pool of nitrogen [12]. Although compatible solutes are thought to maintain cellular homeostasis, there is no consensus yet regarding their role and mechanism of regulation *in vivo* in plants, especially during drought. What is known is that exogenous application of the above amino acids and their derivatives, improves stress tolerance [13]. It has been argued however that accumulation of solutes could be as a consequence of stress rather than an adaptive response [13, 14].

HSPs and DHNs are broadly expressed in grasses under different stress conditions, such as high light, temperature, salinity, oxidative stress, heat and cold, and play crucial roles in maintaining cellular homeostasis, thereby protecting plants from stress [1, 15–17]. HSPs help maintain protein stability, aiding folding and refolding, and conversely are involved in the degradation of damaged proteins under stress conditions. HSPs are found throughout the cell and play very important roles in maintaining normal protein conformation [16]. Although HSPs have been suggested to have protective roles [16, 17], especially in response to heat stress, their functions in other stress conditions, e.g. drought, is less clear [18]. The levels of the chloroplast HSP60 were maintained in drought stressed *Sorghum bicolor* while those of cytosolic Heat Shock Protein HSC 70 were reportedly induced in *Festuca arundinacea* [18, 19]. However, the roles of other HSP families in relation to drought are unclear.

DHNs are Group II of late embryogenesis abundant (LEA) proteins that are hydrophilic, thermostable and accumulate in the cytosol, nucleus, chloroplast and mitochondria [20]. They are characterised as having three conserved motifs, K, Y and S segments, named based on predominance of the amino acids–Lysine, Tyrosine and Serine respectively [21]. Both classes of proteins–HSPs and DHNs–have been shown to be expressed in sorghum and maize in response to drought [15, 22, 23] and have been suggested to have protective roles in both plants [16, 17].

Amongst widely cultivated crops, the C_4_ grass *Sorghum bicolor* is one of the most drought tolerant species [24]. Having C_4_ biochemistry allows it to maintain photosynthesis at low CO_2_ concentrations, e.g. when stomata are significantly closed. However, this alone does not explain its drought tolerance–its close relative maize has similar C_4_ metabolism but much lower drought tolerance. We showed previously contrasting responses in two sorghum varieties—Samsorg 17 and Samsorg 40 with differing degrees of drought tolerance, specifically in terms of high constitutive sugar content and loss of proteins respectively [19]. We also examined their morphological and physiological responses to drought [19]. We observed that soil in pots containing Samsorg 17 dried slower than those with Samsorg 40 but both varieties lost specific leaf area in response to drought [19]. Samsorg 40 had higher above ground biomass but lost chlorophyll per unit leaf area with increasing drought in contrast to Samsorg 17. Samsorg 17 maintained relative water content, open stomata, net assimilation and photosystem II efficiency longer than with Samsorg 40 [19].

In the present study, biochemical and metabolic responses to drought in these two sorghum cultivars are compared using a non-targeted metabolomic approach to gain a fuller understanding of the metabolic differences between varieties. We focussed on identifying the specific sugars accumulated by Samsorg 17 and the nitrogen sinks for lost proteins in Samsorg 40. The two cultivars are also shown to differ substantially in their expression of HSPs and DHNs and in their accumulation of metabolites. The results are discussed in terms of identifying potential targets for developing better drought tolerance.

## Results

### Changes in metabolites during drought

**Fig 1:**
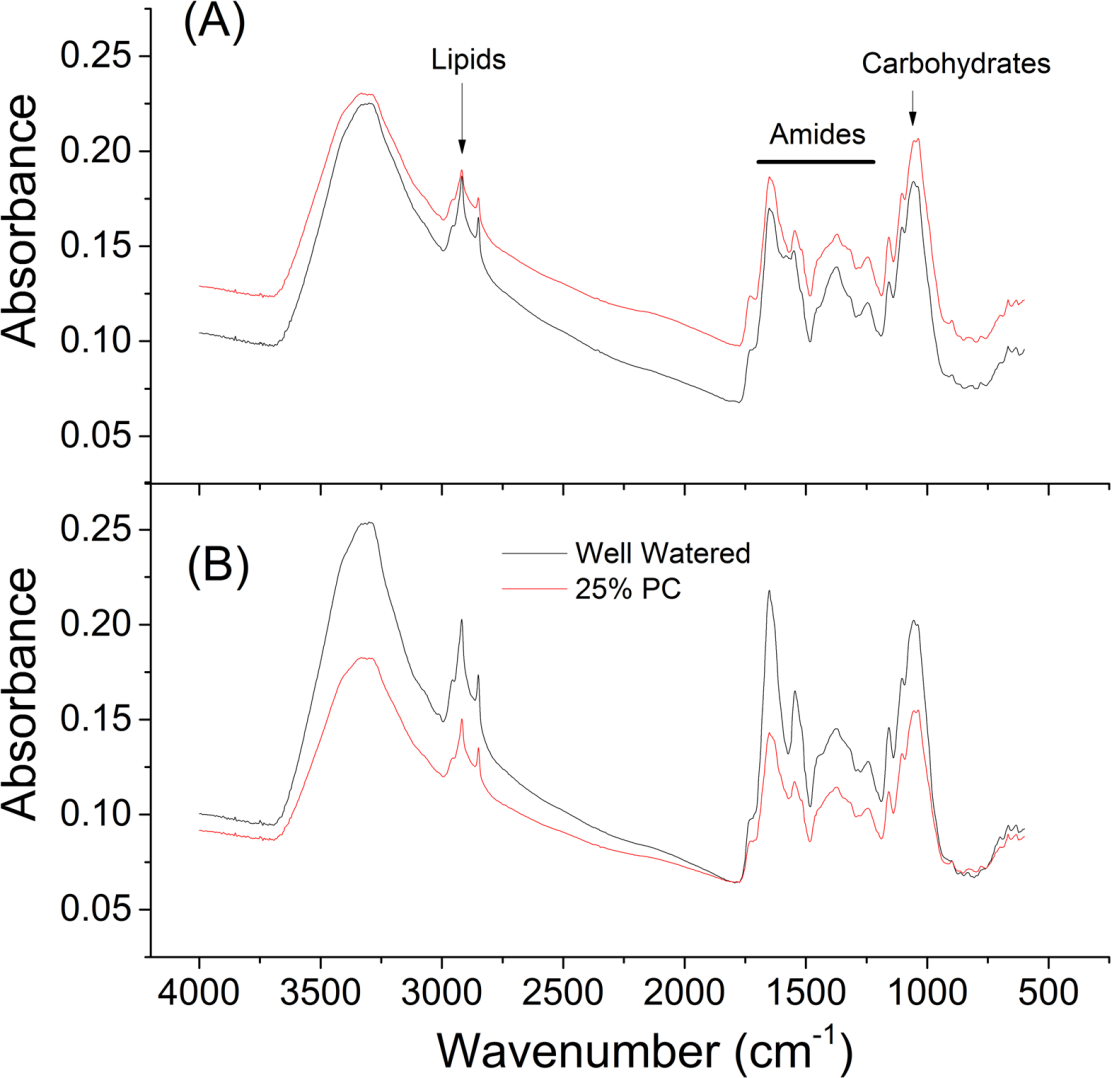
Example FT-IR Spectra. (A) Samsorg 17. (B) Samsorg 40. Extracts from well-watered plants (black lines) or plants exposed to 25% pot capacity (red lines) for up to 5 days. FT-IR raw data were plotted in Origin Pro 8 (OriginLab, Northampton, MA).

Plant pots were allowed to dry to a range of different water contents and then maintained at those levels of dryness for five days, by re-watering pots daily with water lost in the previous 24 h, using an experimental approach described previously [19]. Leaf samples were collected 8h into photoperiod and flash frozen. Ground leaf material was then analysed using FT-IR analysis From this analysis, it can be seen that there are clear differences in chemical composition between cultivars even under control conditions, with differences in the carbohydrate, lipid and amide regions (Fig 1).

**Fig 2:**
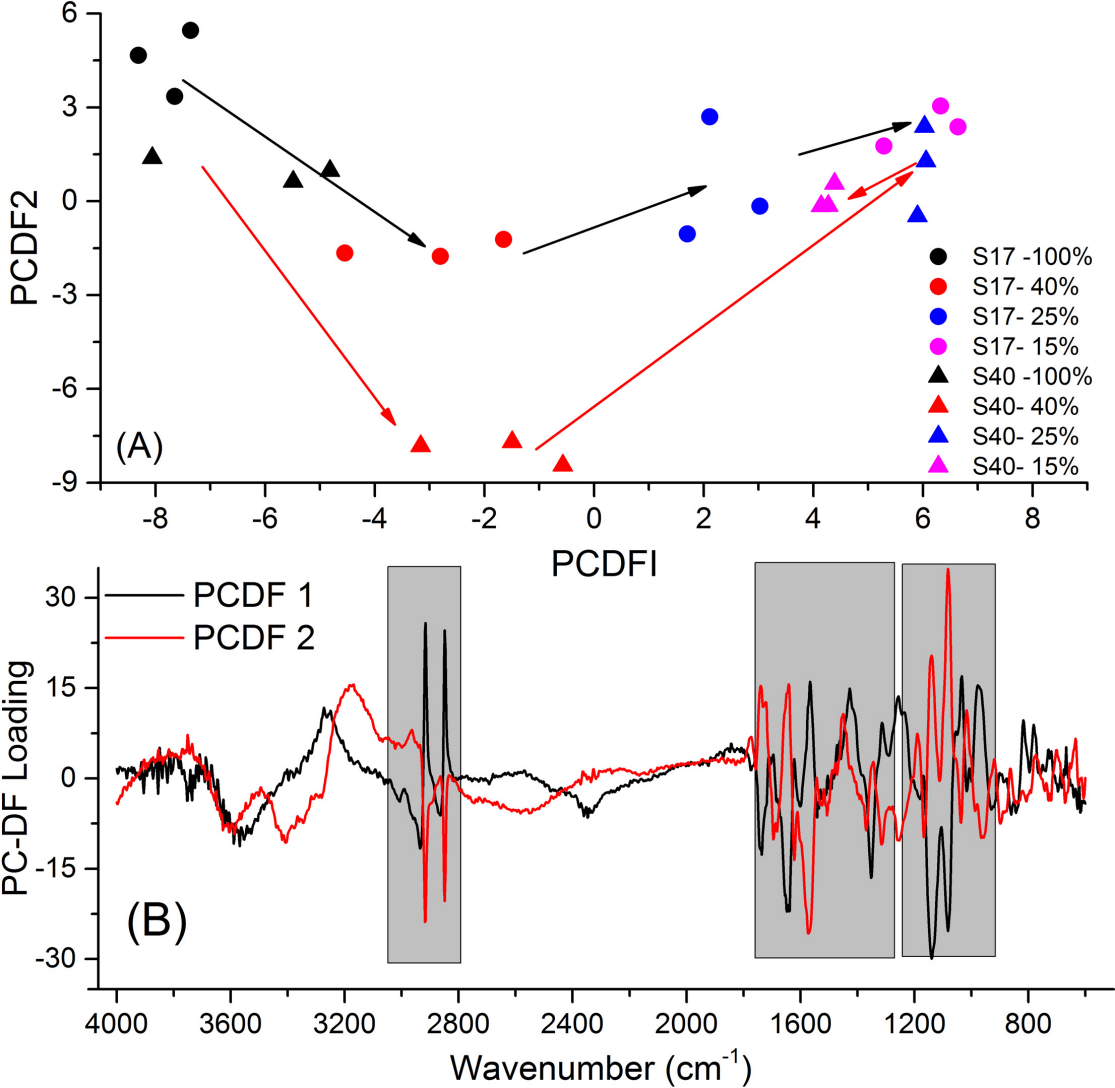
Principal components discriminant function analysis. (A) Scores. (B) Loadings plots of Samsorg 17 and Samsorg 40 acquired using FT-IR spectroscopy. Acquired FT-IR raw data were converted into principal components using the R statistical package. Principal component discriminant function analysis (PC-DFA) was performed on the principal components of the data. The generated discriminant functions were plotted using Origin Pro 8 (OriginLab, Northampton, MA). The shaded regions (from right to left) represent changes in carbohydrates, amides and lipids. Coding used was ‘S17’ or ‘S40’ for Samsorg 17 and Samsorg 40, respectively, and ‘-X%’ refers to percentage SWC.

Principal component-discriminant function analysis (PC-DFA) was used to define key differences in FT-IR spectra. FT-IR spectra showed discrimination between plant lines under control conditions and with differing degrees of drought (Fig 2A). In well-watered plants the two parent lines were well separated. With increasing water deficit, there is variation between the samples that is reflected mainly in PC-DF2. Drying to 40% resulted in a substantial change in both plants, with this being most evident in Samsorg 40. This change could be described by both PC-DF2 and, to a lesser extent PC-DF1. Further drought resulted in a tendency for changes in PC-DF2 to reverse in Samsorg 40, but less so in Samsorg 17, with further changes being described by PC-DF1. Under the most extreme drought conditions, there was a tendency for the two lines to converge. Examination of the PC-DF loadings indicated that changes in carbohydrates, amides and lipids all contributed to both PC-DF1 and 2 suggesting that these are affected by drought (Fig 2B).

### Changes in glucose and sucrose in response to drought

**Fig 3:**
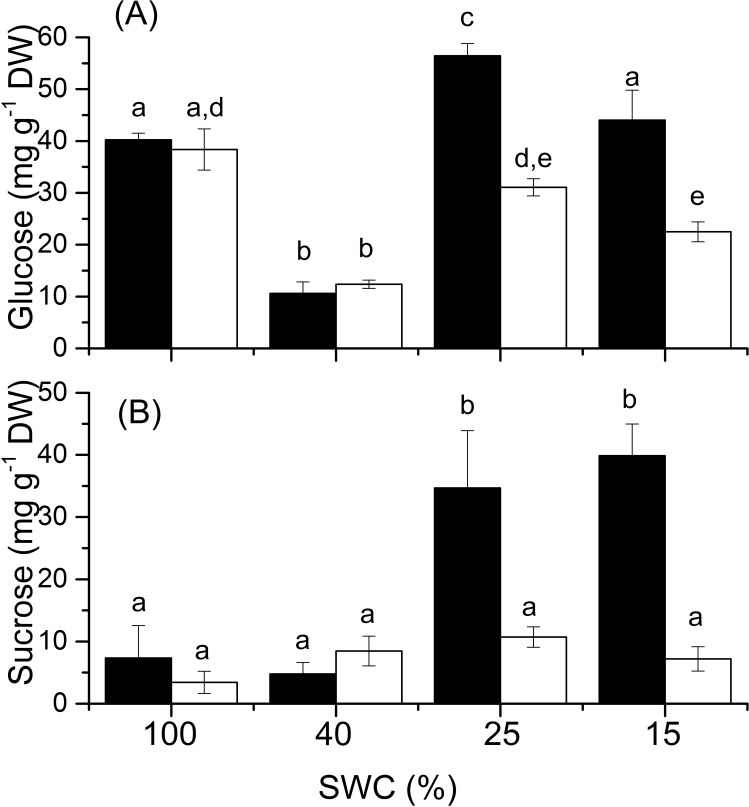
Responses of the soluble sugars. (A) Glucose. (B) Sucrose to drought. Black bars are plants of Samsorg 17 while open bars are those of Samsorg 40. Data are mean of at least 3 replicates and error bars are ± 1 SE. Different letters represent significant difference; ANOVA, *p* < 0.05.

PC-DFA of FT-IR data indicated the occurrence of changes in signals associated with carbohydrates in response to drought in both sorghum varieties. Total soluble sugars were shown previously to be present at high concentrations constitutively in the tolerant Samsorg 17 whilst being induced in response to drought in Samsorg 40 [19]. Sorghum, especially sweet sorghum, is known to accumulate sucrose [25, 26]. Therefore, to determine whether the differences in sugar content between the two varieties studied here was due to sucrose, this was assayed using a linked enzyme assay, which also allowed us to estimate glucose content. When exposed to mild drought (40% pot capacity), there was a transient decrease in glucose content in both cultivars, with the glucose content recovering at more severe drought levels. This effect was greater in Samsorg 17, where glucose content peaked at a concentration significantly above control levels (Fig 3A). Both cultivars showed a further loss of glucose at the highest level of drought, compared to milder conditions. In Samsorg 17, there was a progressive accumulation of sucrose with increasing levels of drought. In Samsorg 40, there was no significant accumulation of sucrose. Previously, we observed that the former contained high concentrations of soluble sugars constitutively, whilst the latter accumulated sugars under drought conditions [19]. This suggests that sucrose accumulation is not making a dominant contribution to the total sugar pool or that other sugars are changing in parallel with changes in sucrose and glucose. In an attempt therefore to identify further sugars as well as other metabolites that might accumulate under drought in the two varieties, plant material was analysed using non-targeted GC-MS analysis.

### GC-MS analysis of differences between Samsorg 17 and Samsorg 40

In this non-targeted metabolomic profiling analysis [27] a total of 188 compounds were detected in the two sorghum varieties using GC-MS, of which 142 were at least tentatively identified (S1 Table. Complete list of compounds detected by GC-MS).

**Fig 4:**
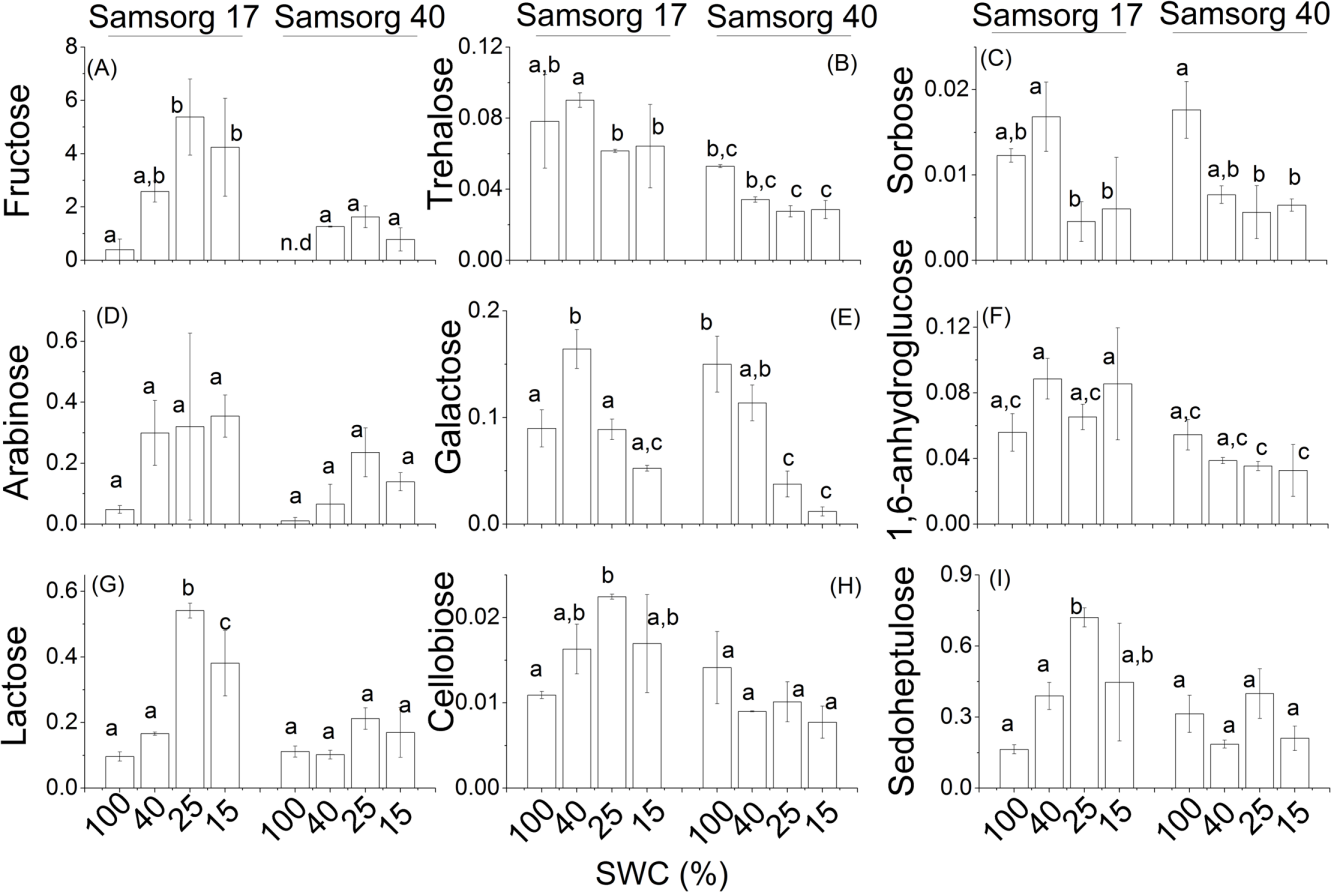
Differences in sugars in the two sorghum varieties. For each panel, the responses of Samsorg 17 plants are shown first at four levels of drought followed by those of Samsorg 40. Data are mean of at least 3 replicates and error bars are ± 1 SE. Data were normalised to Glycine-*d*_5_. Different letters represent significant difference; n.d, not detected; ANOVA, *p* < 0.05.

GC-MS revealed that the drought tolerant Samsorg 17 contained, in addition to glucose and sucrose, significant amounts of fructose, galactose, lactose, cellobiose and sedoheptulose, relative to Samsorg 40. The former also made more sorbose and a compound putatively identified as trehalose, but similar amounts of arabinose and 1,6-anhydroglucose compared with the latter (Fig 4).

**Fig 5:**
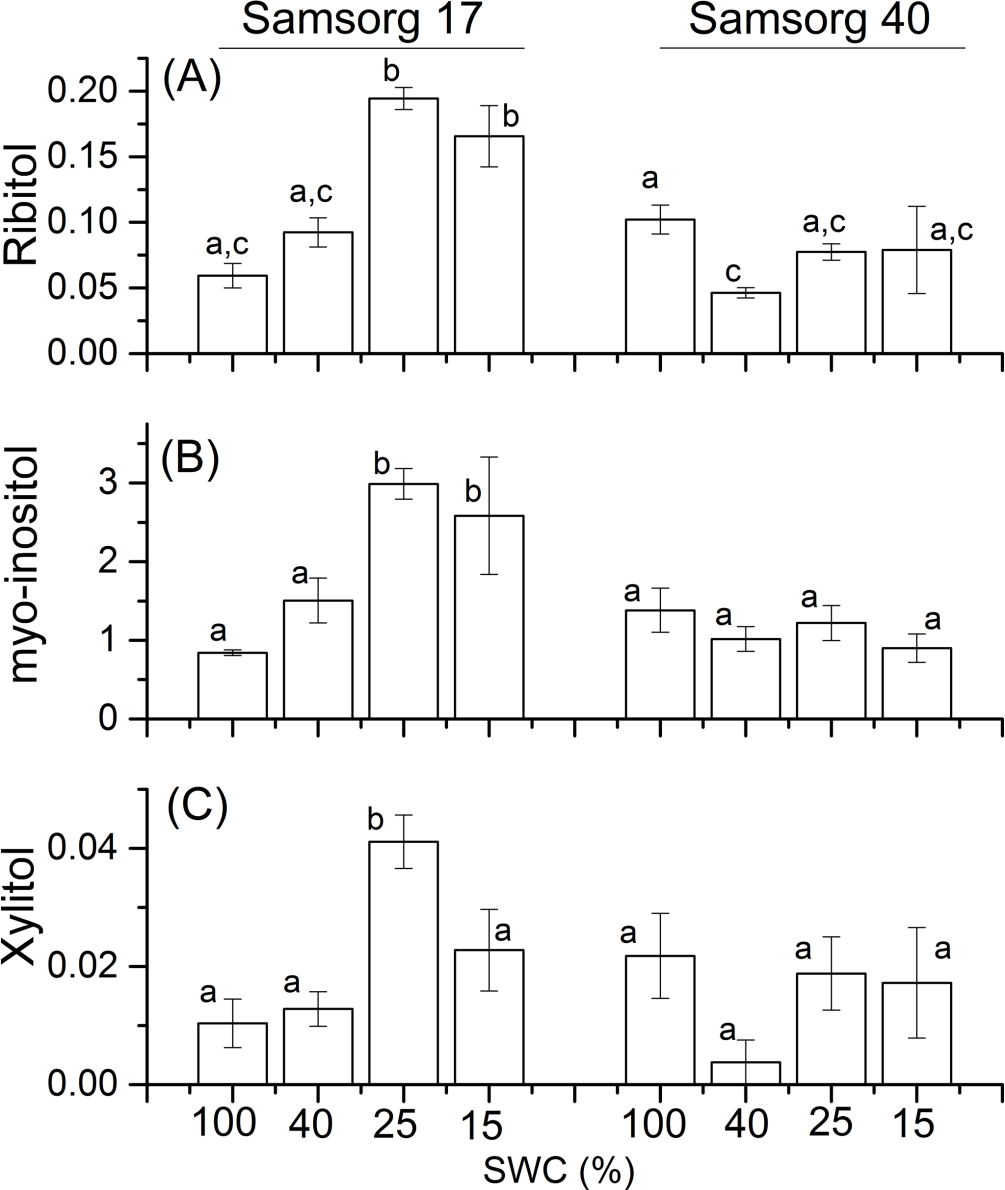
Differences in sugar alcohols in the two sorghum varieties. For each panel, the responses of Samsorg 17 plants are shown first at four levels of drought followed by those of Samsorg 40. Data are a mean of at least 3 replicates and error bars are ± 1 SE. Data were normalised to Glycine *d*_5_. Different letters represent significant difference; ANOVA, *p* < 0.05.

Sugar alcohols have been suggested to play a role in drought tolerance, by acting as osmoprotectants, hence are thought to induce osmotic adjustments [28, 29]. The sugar alcohols–ribitol, *myo*-inositol and xylitol–were significantly accumulated in Samsorg 17 but not in Samsorg 40 in response to drought (Fig 5).

**Fig 6:**
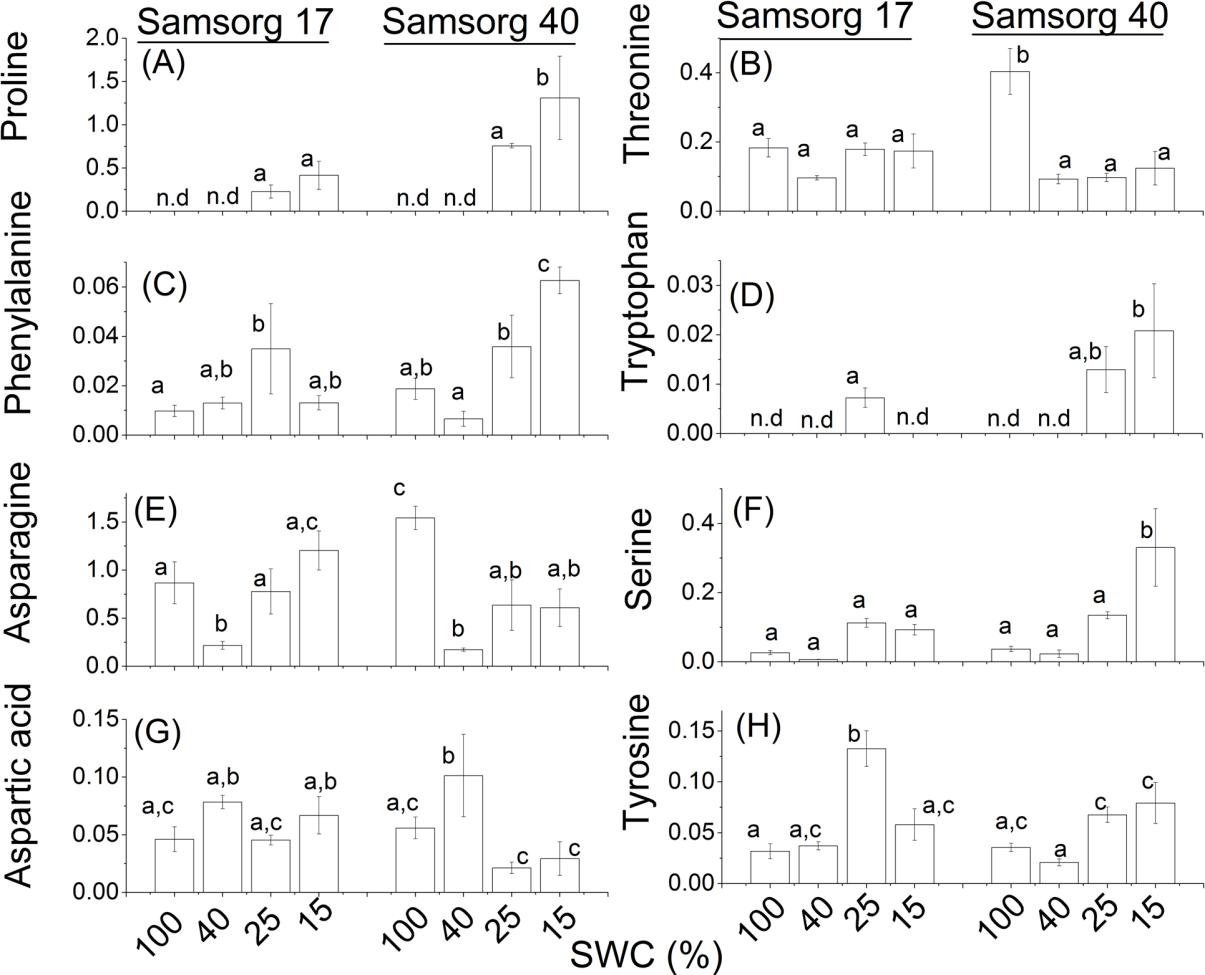
Differences in amino acids in the two sorghum varieties. For each panel, the responses of Samsorg 17 plants are shown first at four levels of drought followed by those of Samsorg 40. Data are mean of at least 3 replicates and error bars are ± 1 SE. Data were normalised to Glycine *d*_5_. Different letters represent significant difference, n.d; not detected; ANOVA, *p* < 0.05.

The osmoprotective role of amino acid accumulation during drought tolerance has been widely studied. It is thought that the accumulation of amino acids can remove reactive oxygen species formed in the cell, thereby improving tolerance [30, 31]. In our study, the less drought tolerant Samsorg 40 accumulated the amino acids proline, phenylalanine, tryptophan, and serine in response to drought, relative to Samsorg 17 (Fig 6).

Threonine and asparagine were however higher under well watered conditions in Samsorg 40. The only amino acid that was accumulated in higher proportions in Samsorg 17 was tyrosine.

**Fig 7:**
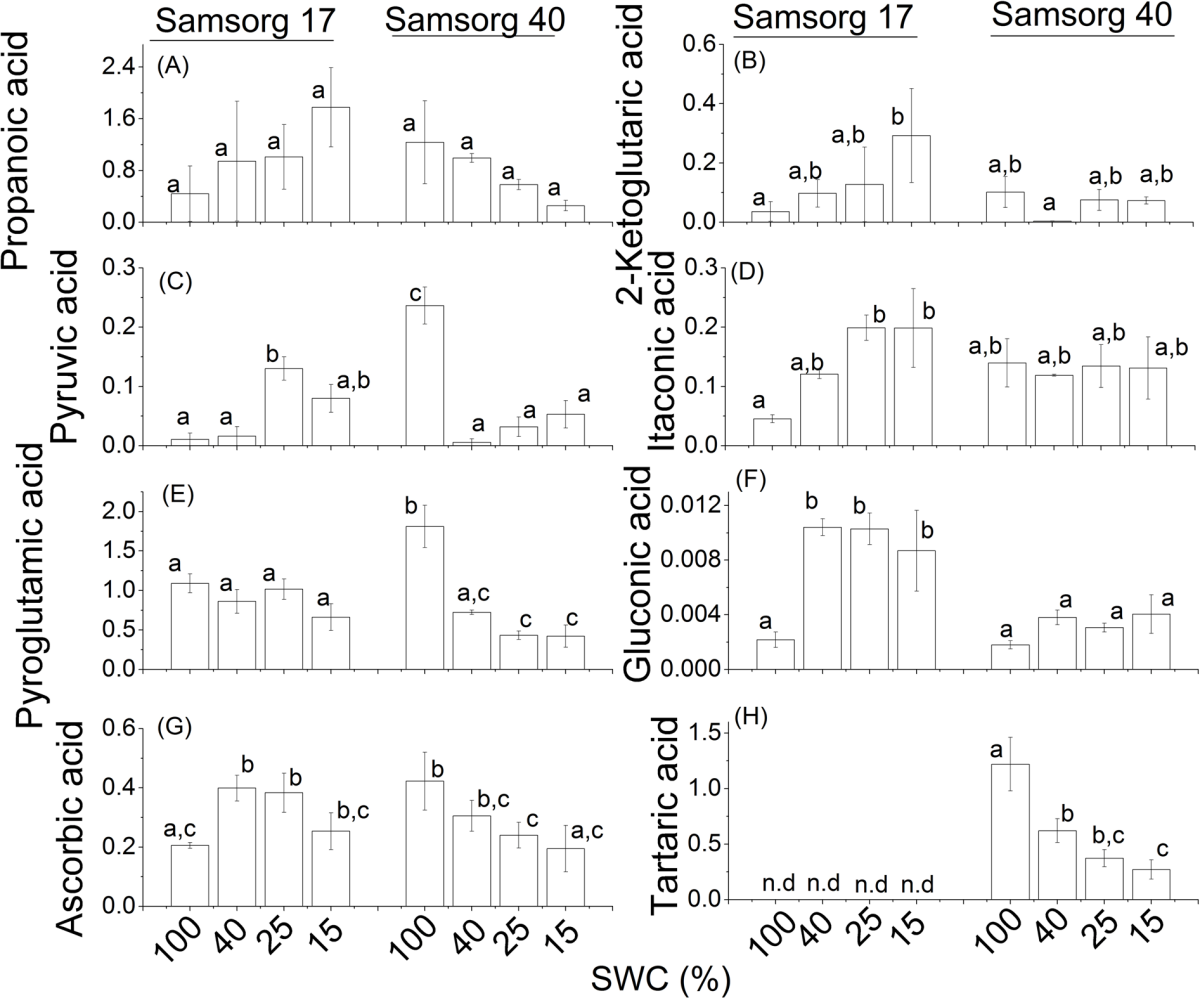
Differences in other organic compounds in the two sorghum varieties. For each panel, the responses of Samsorg 17 plants are shown first at four levels of drought followed by those of Samsorg 40. Data are mean of at least 3 replicates and error bars are ± 1 SE. Data were normalised to Glycine *d*_5_. Different letters represent significant difference; n.d, not detected; ANOVA, *p* < 0.05).

Various organic acids were detected in the sorghum leaves. Ascorbic acid, an important antioxidant, has widely been reported to be induced by stress and is believed to offer some degree of tolerance [32–34]. We found that ascorbic acid did not differ significantly when compared between the two sorghum lines. Amongst the other organic acids detected were propanoic, 2-ketoglutaric, pyruvic, itaconic and gluconic acids. These organic acids also did not differ significantly between the cultivars. The amount of pyroglutamic acid detected was higher under well-watered condition in the less tolerant Samsorg 40 but with drought, dropped to the level seen in Samsorg 17. Tartaric acid was not detected in any treatments in Samsorg 17 but was present in significant amounts in Samsorg 40 dropping in concentration with drought (Fig 7).

### Changes in Heat Shock Proteins

Previous reports have shown that the chloroplast localised 60-kDa HSP, known as chaperonin Cpn60, acts as the Rubisco binding protein (RBP) involved in the folding and assembly of the newly synthesised Rubisco subunits to produce the native enzyme [15–17, 35]. In the leaves of Samsorg 40, Cpn60 declined in parallel with RbcL degradation [19]. Levels of this peptide were maintained in Samsorg 17 throughout the drought period [19]. In order to determine whether the response of HSP60 was typical of other HSPs, we examined other HSP families—HSP70, HSP90 and HSP101.

Chloroplast localised chHSP70 is thought to be involved in protein import and translocation processes in chloroplasts. It also participates in the repair of damaged PSII proteins, by promoting either synthesis or assembly of new reaction center components [36–38]. chHSP70 was significantly up-regulated from 40% PC in Samsorg 17. In Samsorg 40, a similar up-regulation was observed but this effect was less marked.

**Fig 8:**
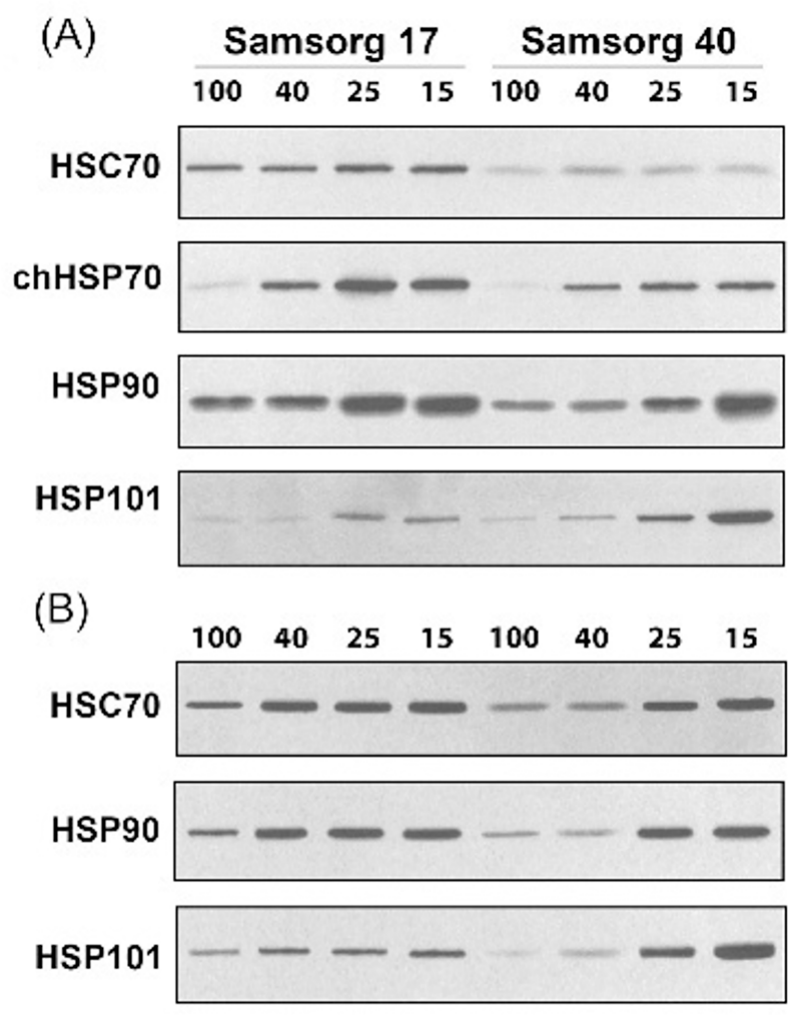
Heat Shock Protein expression. (A) In the leaf. (B) root of Samsorg 17 and Samsorg 40. Plants were maintained at four different water levels -100, 40, 25 and 15% of pot capacity for 5 days. Equal amounts of whole leaf and root proteins (25 μg) were separated using electrophoresis on Glycine-SDS-PAGE (4–20%), electroblotted onto nitrocellulose membrane, and probed with sets of antibodies raised against HSP chaperone proteins. Blots are representative of at least three independent experiments.

Chloroplast localised HSPs were not detected in root tissues (Fig 8). The cytosolic protein, Heat Shock Cognate 70 (HSC70) ensures correct folding of polypeptide chains to proteins [17]. HSC70 was constitutively expressed in leaves of both Samsorg 17 and Samsorg 40, however the level of expression appeared stronger in the former than the latter (Fig 8A). There was no clear indication that this expression was affected in response to drought in these tissues. In roots, there was some indication that HSC70 was induced by drought, especially in Samsorg 40 (Fig 8B). Cytosolic HSP90, a molecular chaperone that only interacts with well-folded proteins was up-regulated in response to drought in leaves and roots of both cultivars, while Samsorg 17 appeared to show stronger expression than Samsorg 40. HSP 101, which belongs to a family of heat shock proteins named HSP 100s (or Clp proteins) and functions in protein disaggregation and degradation, accumulated in leaves during the late stages of drought in both lines, but considerably more so in Samsorg 40 (Fig 8A). A similar pattern was observed in roots of Samsorg 40, whilst in Samsorg 17 a more even expression pattern was seen with differing degrees of drought (Fig 8B).

### Dehydrin/ Dehydrin-like protein expression

**Fig 9:**
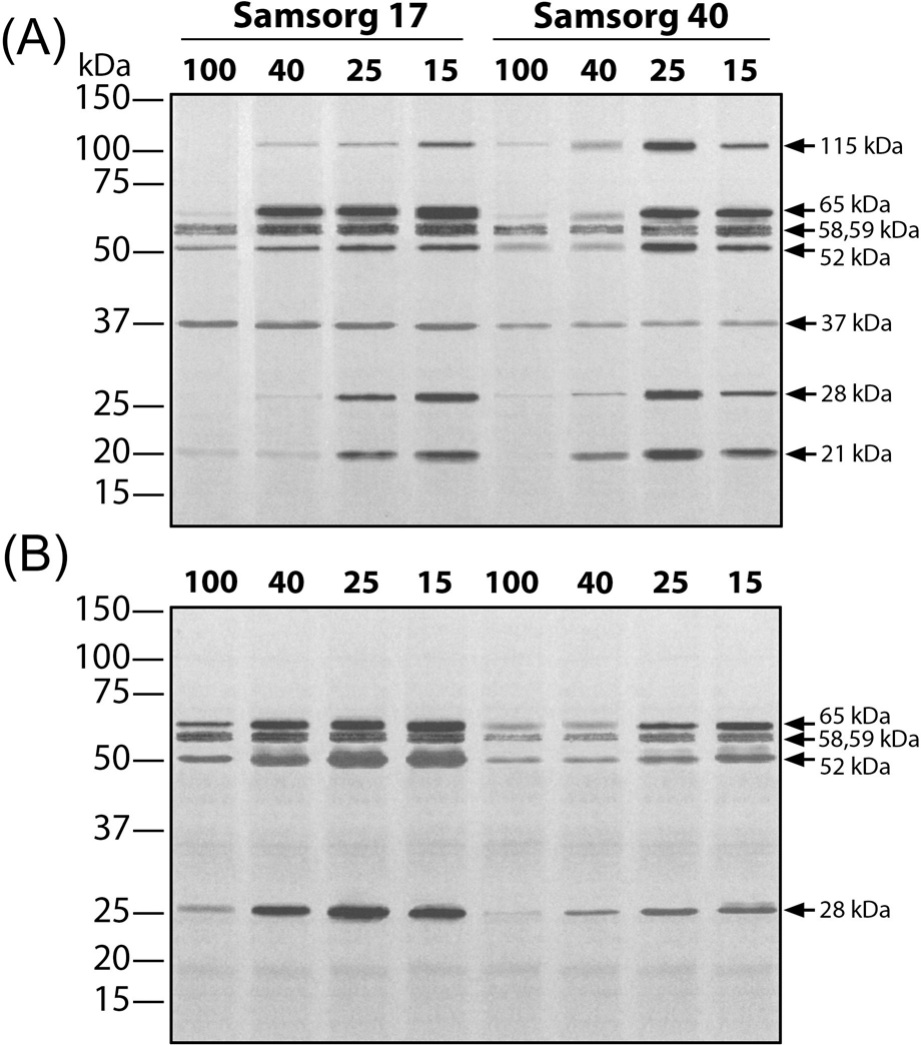
DHN/ DHN-Like protein expressions in *Sorghum bicolor* L. (var. Samsorg 17 and Samsorg 40) under drought treatments-100 (Control), 40, 25 and 15% of pot capacity. Equal amounts of whole leaf (A) and root proteins (B) (25 μg) were separated on Glycine-SDS-PAGE (4–20%), eblotted onto nitrocellulose membrane and probed with set of antibodies raised against the K-segment of DHN. Blots are representative of at least three independent experiments.

Dehydrins are Group II of Late Embryogenesis Abundant (LEA) proteins. They have a conserved lysine rich amino acid sequence and are thought to contribute to stress tolerance [21, 39]. Samsorg 17 and Samsorg 40 showed contrasting DHN/ DHN-like protein expression when subjected to differing degrees of drought. In leaf tissue, peptides reacting to a DHN antibody ranged in size from 21 to 115 kDa. Amongst these, different expression patterns could be seen. Fewer bands were observed in roots, with bands at approx. 115, 37 and 21 kDa being absent. The band at approx. 37kDa was constitutively expressed in the leaves of both cultivars; however bands appeared more intense in Samsorg 17 than Samsorg 40 (Fig 9A). Other bands showed more or less clear induction in response to drought. Bands in the range 52–69 kDa were observed in both cultivars and were induced in response to drought in both shoots and roots; however these tended to be induced sooner and/or more strongly in Samsorg 17 than in Samsorg 40. In contrast, the leaf only bands at 21 and 115 kDa showed earlier and stronger induction in Samsorg 40. The band at around 28 kDa showed similar expression patterns in roots and shoots of both cultivars, although expression appeared stronger in roots of Samsorg 17 (Fig 9).

## Discussion

To increase the drought tolerance of crop species, we need to understand better the traits possessed by drought tolerant plants so that these can be transferred into new varieties. In this study, two varieties of Sorghum–Samsorg 17, a highly drought tolerant cultivar, and Samsorg 40, a less drought tolerant one–were studied. Our previous results have shown that these varieties differ markedly in their physiological responses to drought, with evidence for strongly contrasted biochemical responses to drought in closely related plants [19]. Results presented here, reinforce those earlier results, and show that Samsorg 17 induced higher levels of heat shock proteins (HSPs) and dehydrins (DHNs) but differential accumulation of metabolites compared with Samsorg 40.

FT-IR analyses the infrared absorption spectra of chemical species that absorb in the mid infrared part of the electromagnetic spectrum [40]. Together with PC-DFA loadings, it is possible to determine what types of molecules are being affected by stress in plants. PC-DFA performed showed clear variation between the sorghum varieties. The sorghum samples were clearly different under well-watered and moderate drought conditions but converged at extreme drought. PC-DFA loadings showed that sugars, amides (from proteins) and lipids are affected by drought in the two sorghum cultivars (Fig 2) corroborating the changes identified on the raw spectra (Fig 1).

Previously, we showed that plants of Samsorg 17 contain significantly higher total sugar content than those of Samsorg 40 [19]. It has been shown that sorghum, especially sweet sorghum, accumulates sucrose in its stems [25, 26] making this a strong candidate to explain the high sugar content of Samsorg 17. Both sorghum varieties accumulate high concentrations of glucose in response to drought but Samsorg 17 accumulates sucrose to significantly higher levels than Samsorg 40. However, neither this accumulation of sucrose nor the concentrations of glucose explain our earlier findings, that total soluble sugar levels were constitutively high in Samsorg 17 whilst high levels were induced by drought in Samsorg 40 [19]. The conditions of drought treatment in our earlier study were different–there drought was allowed to develop progressively, whilst here, plants were maintained at a particular level of drought. Nevertheless, it seems likely that the presence of other sugars, explains the differences between the varieties.

To identify other sugars accumulating in the two lines, leaf samples were analysed using GC-MS. PCA revealed a distinct response at lower levels of drought in the two cultivars (S1 Fig) Typically the sugars quantified in this way showed a similar behaviour to sucrose, tending to be present at higher concentrations in Samsorg 17 and being induced to a greater extent in response to drought. Increases in fructose, lactose, cellobiose and sedoheptulose were induced by drought in Samsorg 17 (Fig 4). There were also apparent higher levels of the monosaccharides–galactose, 1, 6-anhydroglucose, sorbose, arabinose, and a disaccharide tentatively identified as trehalose in Samsorg 17 (Fig 4). This is consistent with high sugar content or sugar accumulation playing a crucial role in maintaining leaf function and photosynthesis during drought, as reported earlier [5, 41, 42]. These data do not explain our previous finding that Samsorg 17 stores high constitutive amounts of soluble carbohydrate, suggesting that other sugars or related compounds, not detectable are important in Samsorg 17. Sugar alcohols also increased significantly in Samsorg 17 suggesting that these may have a role as osmoprotectants and may contribute to the stabilization of protein structure against degradation (Fig 5) [11, 28, 29]. Overall, higher accumulation of soluble sugars and sugar alcohols in Samsorg 17 suggest that these may play a role in lowering shoot and root water potential for better water uptake.

In addition to differences in sugar accumulation, Samsorg 17 showed significant up-regulation of gluconic acid in contrast to Samsorg 40. Higher level of gluconic acid suggests that it is protective. Ascorbic acid was not markedly different between the cultivars but was induced to a lesser extent with progressive drought in Samsorg 17 relative to Samsorg 40 (Fig 7). Ascorbic acid has antioxidant properties and is thought to offer some degree of tolerance to plants. The effects of many forms of environmental stress are known to be mediated, at least partially, by an enhanced generation of reactive oxygen species (ROS) in the organelles with highly oxidizing activities and sustained electron flows–chloroplasts, mitochondria and peroxisomes [43, 44]. A cellular system to scavenge ROS, including the low-molecular-weight antioxidants such as ascorbate, may protect membrane lipids and proteins from damage [32–34]. Although ascorbate was not clearly different in the two sorghum lines with drought in this study, these results are consistent with the high, stress-induced levels of ascorbate found in both the mesophyll and bundle sheath chloroplasts isolated from Samsorg 17 compared with Samsorg 40 (Stepien et al, unpublished data). The roles of the other organic acids detected are not clear; however, some of them are speculated to be imported and exuded from the root system which could improve an acquisition of the mineral elements under drought conditions.

It is generally accepted that the uptake of nutrient by crop plants is reduced in dry-soil conditions. Drought provokes nutritional deficiencies, even in well-fertilized environment, as the physiochemical properties of soil may reduce availability of individual nutrients. In addition, any negative effect of drought on the mineralization process of organic matter depending on the soil microorganisms and enzymes activity, affects the amount of inorganic nutrients [45, 46]. On the other hand, drought may also increase the allocation of resources and increase production of root exudates, contributing to increased chelation and uptake of nutrients [47, 48]. Organic acids are an important class of exudates and their release to the rhizosphere contributes to plant health in several ways, including mobilization of the unavailable forms of essential nutrients. The organic acids detected by GC-MS are believed to be the ones released from the roots of the plants. These acids may solubilise P, Fe, Mn and/or Zn from soil [47].

Previously we observed an accumulation of the amino acid proline and a loss of soluble protein in plants of Samsorg 40 exposed to progressive drought. Here (Fig 6), examining plants exposed to sustained drought, we observed the same accumulation of proline, consistent with a large accumulation of this amino acid, which is widely recognised as playing a role in drought tolerance. This increase in proline is also seen in Samsorg 17, but to a much lower level, again consistent with our previous results. The accumulation of proline in Samsorg 40 is also accompanied by the accumulation of most other amino acids measured (Fig 6).

These metabolic alterations were accompanied by clear changes in the abundance of protein present in the two sorghum lines [19]. Amongst these, changes were noted in the various HSPs and DHNs. These proteins have previously been characterised as being expressed in grasses in response to various stress conditions including light, temperature, salinity, oxidative stress, heat and cold [1, 15–17]. However, little is known about the roles of these proteins in response to drought. What is known is that the maintenance of chloroplast localised chaperonin (Cpn60) and the cytosolic Heat Shock Protein (HSC70) offer some level of drought tolerance [15, 18, 19, 22, 23].

Amongst the HSPs examined, chloroplast localised chHSP70, the constitutively expressed HSC70 and HSP90 all showed either higher apparent expression and/or induction at a lower level of drought in Samsorg 17 than Samsorg 40 (Fig 8). chHSP70 participates in PSII repair process by promoting either synthesis or assembly of new reaction center components [37]. Greater expression of chHSP70 in Samsorg 17 might be responsible, at least partially, for the maintenance of PSII protein complex and PSII electron transport under stress conditions [19]. HSC70 was constitutively expressed in both cultivars, albeit higher in Samsorg 17 (Fig 8). HSC70 is a house-keeping protein that is constitutively expressed in the cytosol [49]. It prevents protein aggregation and hence ensures correct folding of polypeptide chains to proteins [17]. This probably contributed to the maintenance of protein structure in Samsorg 17, which improved the tolerance of the plant. HSP90 is another HSP that was accumulated in higher level in the more tolerant Samsorg 17 relative to Samsorg 40. HSP90 is a molecular chaperone that only interacts with well-folded proteins. It is distinct from many other molecular chaperones in that most of its known substrates are signal transduction proteins such as hormone receptors and signaling kinases. HSP90 only functions when large protein complexes involving co-chaperones such as chHSP70 have induced folding in the chloroplast [16, 17]. The apparent up-regulation or earlier induction of HSP90 in the leaves and roots of Samsorg 17 is consistent with their role being to enhance the drought tolerance and continued survival of these plants under drought stress. In contrast HSP101, a family member of the Clp heat shock proteins, was the only HSP examined where apparent expression was higher in Samsorg 40, with only trace levels of this protein being detectable in leaves of Samsorg 17. Rather than the regular chaperone function of preventing protein aggregation and misfolding, this family functions in protein disaggregation and degradation [16, 17]. The greater induction of this protein in Samsorg 40 is consistent with damage being induced and with damaged proteins having to be degraded. Curiously however, a comparatively higher level of expression was seen in the roots of Samsorg 17, possibly reflecting an earlier reaction to drought in roots.

We found a generally higher induction of DHNs in Samsorg 17 than Samsorg 40 (Fig 9). The functions of dehydrins (DHNs) are still under investigation, however the relationship between DHNs and drought has been examined [50]. They are characterized by a high content of glycine, threonine, asparagine, glutamine, serine, aspartic and glutamic acids, explaining their highly hydrophilic nature [21, 39, 51]. It is believed they are induced by ABA during drought [50, 52]. They are known to be thermostable and hence are thought, like HSPs, to have chaperone-like properties and roles in membrane structure stabilization [20, 53]. The higher expression levels and earlier induction of these proteins in Samsorg 17 is again consistent with their playing a role in maintaining cell function for longer in response to drought.

In conclusion, we have observed that Samsorg 17 and 40 show contrasted responses to drought. In the drought tolerant Samsorg 17, there was a general up-regulation of protective proteins- HSPs, DHNs- sugars and sugar alcohols. In contrast, Samsorg 40, the less tolerant sorghum cultivar, showed a lower accumulation of potentially protective metabolites, but did accumulate significantly more of various amino acids, probably linked to the breakdown of protein previously reported [19]. The up-regulation and higher levels of sugar molecules, in particular high amount of fructose, in Samsorg 17, suggest that they play protective roles in maintaining leaf function and possibly maintaining water content during water deficit. The mobilisation of N compounds in Samsorg 40, may allow movement of N to young tissues for possible recovery. In the future, it would be useful to examine contributions of these different responses, for example using recombinant inbred lines or a segregation population generated from the two sorghum lines.

## Materials and Methods

### Plant material and growth conditions

Seeds of Sorghum (*Sorghum bicolor* L) cv Samsorg 17 (more drought tolerant) and cv Samsorg 40 (less drought tolerant) were provided by the Institute of Agricultural Research, Zaria, Nigeria. The seeds were grown in a growth room as described previously using John Innes No. 3 compost [19]. To estimate relative soil water content (SWC), % maximum pot capacity, fully saturated soil was weighed and thereafter oven dried at 105°C to constant weight. The weight difference between the water-saturated and oven-dried soil was taken as the weight of water needed to bring pots to pot capacity and lower water levels (% pot capacity) estimated accordingly. Plants were allowed to establish for 21 days under well watered conditions before the onset of drought treatments. For droughted plants, watering was stopped and pots allowed to dry until the soil SWC fell to a particular level. Pots were then maintained at different water levels by re-watering daily with water lost in the previous 24 hours, for 5 days before samples were harvested. Samples for metabolomic analysis were harvested by flash freezing leaf material collected 8 h into photoperiod. Samples of leaves and roots for protein analysis were harvested in the same way. For root analysis, prior to sample collection, roots were washed with distilled water to remove debris. 250mg of whole-root soluble proteins were ground into a fine powder in liquid nitrogen and extracted in ice-cold 150mM Tris-Hcl buffer as described previously [19].

### Fourier transform infrared (FT-IR) spectroscopy

Fully expanded leaf and shoot tissues from different soil water contents were excised 8 h into the photoperiod with a pair of scissors, flash frozen in liquid nitrogen and lyophilised (freeze-dried) with a ScanVac Coolsafe freeze dryer (Vacuubrand, Wertheim, Germany) for 48 hr. FT-IR spectroscopy was performed on 30 mg DW of tissues homogenised, transferred to 2 mL microcentrifuge tubes and extracted in 600 μL of distilled water. 5 μL of homogenate was loaded onto the wells of a silicon 96 target plate (Bruker, MA, USA) and dried at 60°C. The Si plate was then placed in a Bruker Equinox-55 spectrometer and raw FT-IR data recorded in the absorbance mode of wavenumber 4000–600 cm^-1^ with a resolution of ~4 cm^-1^.

After data generation principal components analysis (PCA) was used to analyse the data and this was performed in the R software environment (available at http://www.r-project.org/). PCA scores and loadings plots were generated in Origin Pro 8 (OriginLab, Northampton, MA). Principal component discriminant function analysis (PC-DFA), a supervised multivariate technique was performed on the principal components (PCs) of samples harvested at different soil water contents. This was performed to determine the spectral regions of compounds (from PC-DFA loadings plots) that change with water deficit. PC-DFA is used to discriminate between samples based on their principal components with knowledge of which spectra were replicates; in this case 8 groups were used for the 2 plants × the 4 SWC conditions. For clarity, the spectral regions of major compound classes are found for lipids at ~ 3050–2800 cm^-1^, proteins at ~ 1750–1250 cm^-1^ and carbohydrates at ~ 1250–900 cm^-1^ [54–56].

### Gas Chromatography-Mass Spectrometry (GC-MS)

Fully expanded leaf and shoot tissues from plants exposed to different soil water contents were excised 8 h into the photoperiod with a pair of scissors, flash frozen in liquid nitrogen and lyophilised (freeze-dried) with ScanVac Coolsafe freeze dryer (Vacuubrand) for 48 h. 30 mg dry weight (DW) of tissues were extracted in methanol-chloroform-water mixture as described in Lisec, Schauer (57) and Allwood, Ellis (55). Polar phase extracts were dried using a speed vacuum concentration with 100 μL of an internal standard containing 200 μg/mL of succinic-*d*_4_ acid, lysine-*d*_4_ and glycine-*d*_5_ (Sigma-Aldrich, St Louis, Missouri, USA). This was followed by a two-step derivatisation process according to Fiehn, Kopka [58], Lisec, Schauer [57] and Allwood, Ellis [55], which involves first the addition of methoxyamine hydrochloride dissolved in pure pyridine (Sigma-Aldrich) and second, treatment with a silylating compound *N*-methyl-*N-*(trimethylsilyl)trifluoroacetamide (MSTFA reagent; Macherey-Nagel, Duren, Germany). Subsequently, 20 μL of a retention index (RI) mixture was also added containing 3 mg/mL of docosane, nonadecane and 3 μL/mL decane, dodecane, pentadecane. Samples were then analysed using a Leco Pegasus III (4D) GC x GC/MS in GC/MS mode (Leco Corp., St Joseph, MO) in conjunction with a Gerstel MPS-2 autosampler (Gerstel, Baltimore, MD) and an Agilent 6890N gas chromatograph (Agilent Technologies, Santa Clara, CA, USA). Within the system, an electron ionisation (EI) source was employed for ion formation/fragmentation and mass detection was conducted using a time-of-flight (TOF) mass analyser. Quality control (QC) samples were obtained by mixing equal volumes of the upper polar phase of all the sample extracts and were analysed after every 5^th^ sample. The retention time and/or retention index of metabolites identified were compared and matched with those from pure chemical standards using in-house libraries from the Manchester Metabolomics Database [55]. The GC-MS data was aligned using an in-house LOESS alignment algorithm and missing values using a KNN infill within Matlab. The PCA (using PCs 1 & 2) was plotted with Simca-P version 11 (Umetrics AB, Umea, Sweden).

### SDS-PAGE and Immunoblot analysis

Protein extraction and chloroplast isolation were performed as described previously [1919]. Protein samples for SDS-PAGE were incubated in 50 mM Tris-HCl (pH 6.8), containing 2% (w/v) SDS, 50 mM DTT, 1 mM PMSF, 5 mM EDTA, 10% (w/v) glycerol and 0.05% (w/v) bromophenol blue at 95°C for 3 min. Total protein from Sorghum leaf/root (25 μg) or chloroplast (15 μg) was separated on 4–20% Precise Tris-Glycine SDS-PAGE (Thermo Scientific, Waltham, MA, USA) in Mini-PROTEAN 3 cell (Bio-Rad, CA, USA) at 35 mA per gel. For immunoblot analyses, proteins were transferred to Hybond-ECL nitrocellulose membrane (Amersham, Little Chalfront, UK) by electroblotting in western transfer buffer (25 mM Tris-HCl, pH 8.3, 192 mM glycine and 20% (v/v) methanol) in Mini Trans-Blot cell (Bio-Rad; 60 min at 90 V). Blots were checked by Ponceau S staining to ensure even transfer. Membranes were blocked with 5% (w/v) non-fat milk powder in TBS-T (20 mM Tris-HCl, pH 7.6, 125 mM NaCl and 0.5% [v/v] Tween-20) and then incubated overnight at 4°C with primary antibodies against: Dehydrins (Agrisera, Vannas, Sweden). Heat Shock Proteins–HSC/HSP70 (Enzo, Exeter, UK), chHSP70, HSP90-2 and HSP101 (Agrisera, Vannas, Sweden). Dehydrins (DHNs) are Group II of Late Embryogenesis Abundant proteins with conserved motif in C-terminal amino acid sequence termed K-Segment. Anti-DHN antibody raised against this sequence recognises a number of polypeptides in the range 9–200 kDa. Binding of the primary antibody was detected using appropriate horseradish peroxidase (HRP)-conjugated secondary antibodies (1:25000 polyclonal goat anti-rabbit IgG; Sigma) and ECL detection reagent (Amersham) according to the manufacturer's instructions.

### Sucrose and Glucose Assays

100 mg fresh weight (FW) of sorghum samples were weighed and extracted in 1 mL of distilled water. 0.1 mL of sorghum extracts from different soil water contents (% pot capacity) were assayed for sucrose and glucose using Sucrose/D-Glucose assay kit (catalogue number K-SUCGL; Megazyme, Ltd., Bray, Ireland). Assays were performed according to the manufacturer’s instructions. The principle of the kit involves the hydrolysis of sucrose by the enzyme β-fructosidase to D-glucose and D-fructose. Free D-glucose catalysed by glucose oxidase and peroxidase in the presence of *p*-hydroxybenzoic acid and 4-aminoantipyrine was then determined based on the concentration of a red coloured quinoneimine dye. FW results obtained were subsequently converted to DW using the formula: %DW = %FW / [1-(RWC/100)], where DW is dry weight and RWC is relative water content.

### Statistics

A two-way ANOVA (analysis of variance) with a Tukey post-hoc test was conducted using SPSS Statistics 20 statistical software (IBM, Portsmouth, UK), to compare the responses of the two sorghum lines to water deficit. Different letters represent significant differences at *p* < 0.05.

## Supporting Information

S1 Fig Sorghum drought PCA from GC-MS data. B-100, 40, 25 and 15 (Samsorg 17 at 100, 40, 25 and 15% SWC); C-100, 40, 25 and 15 (Samsorg 40 at 100, 40, 25 and 15% SWC); PC1 & 2 (Principal components 1 & 2); QC1–9 (Quality controls 1–9). Plot based on 3 biological replicates at the respective % SWC).
(TIF)

S1 Table Complete list of compounds detected by GC-MS. (XLSX)
