# Supplementary figures and images for: Biochemical Analyses of Sorghum Varieties Reveal Differential Responses to Drought
Source: PLoS One. 2016 May 6;11(5):e0154423. doi: 10.1371/journal.pone.0154423 (PMC4859509; doi:10.1371/journal.pone.0154423)

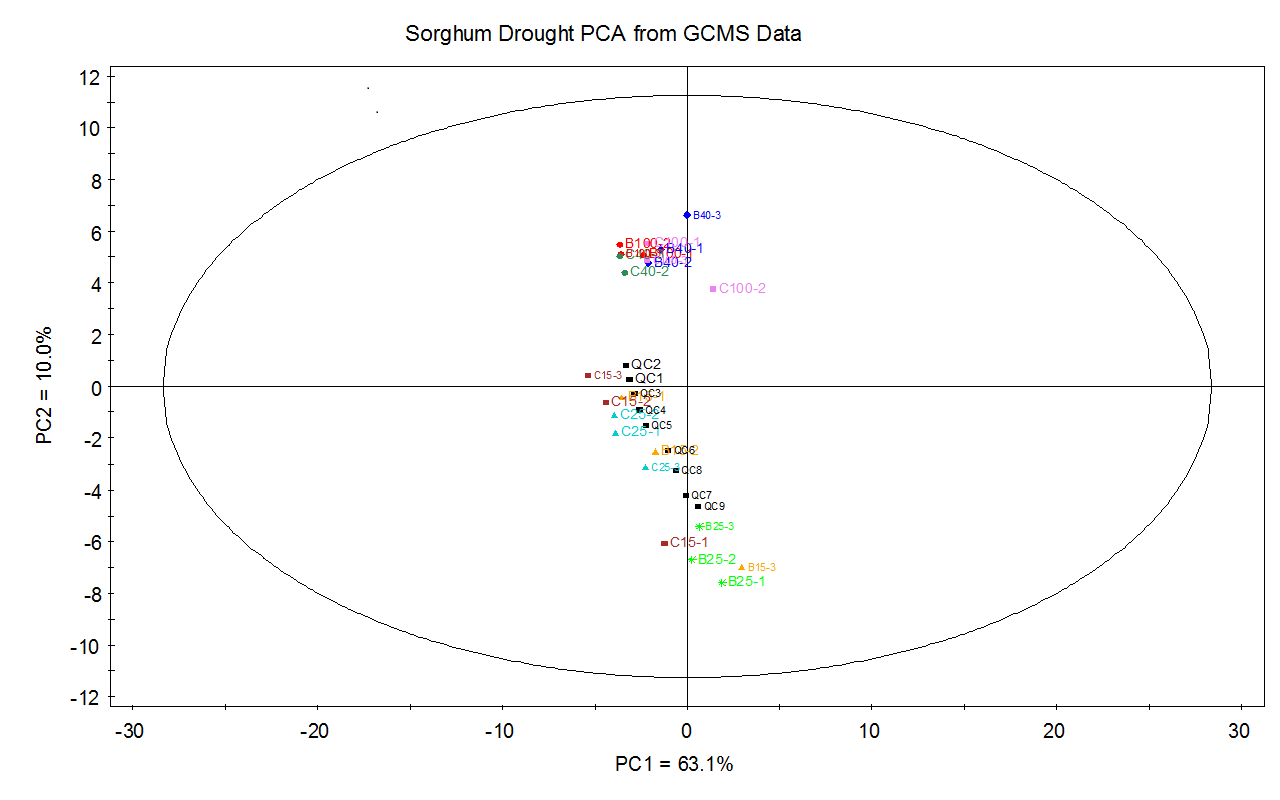

Supplement: S1 Fig — Plot based on 3 biological replicates at the respective % SWC). (TIF) [file pone.0154423.s001.tif]
